# Current Knowledge on Spinal Meningiomas—Surgical Treatment, Complications, and Outcomes: A Systematic Review and Meta-Analysis (Part 2)

**DOI:** 10.3390/cancers14246221

**Published:** 2022-12-16

**Authors:** Victor Gabriel El-Hajj, Jenny Pettersson-Segerlind, Alexander Fletcher-Sandersjöö, Erik Edström, Adrian Elmi-Terander

**Affiliations:** 1 Department of Clinical Neuroscience, Karolinska Institutet, 17177 Stockholm, Sweden; 2 Stockholm Spine Center, Löwenströmska Hospital, 19445 Upplands Väsby, Sweden

**Keywords:** spinal meningioma, surgical treatment, Simpson grade, complications, recurrence, outcomes, health-related quality of life

## Abstract

**Simple Summary:**

Spinal meningiomas are the most common adult primary intradural spinal tumors. There are no recent comprehensive reviews summarizing the current state of the evidence for spinal meningiomas. This review aims to cover all relevant studies on spinal meningiomas published over the past 20 years. Three electronic databases were systematically searched, and 104 studies were identified. Briefly, spinal meningioma surgery is a safe procedure. The most relevant predictors of unfavorable outcome were poor preoperative status, longer time to surgery, and surgery for recurrent tumors. Our analysis revealed that higher WHO grade, higher Simpson grade, ventral tumor location, and male sex were all associated with higher odds of recurrence. Finally, surgery was associated with significant improvements in health-related quality-of-life.

**Abstract:**

Background: Most of the knowledge on spinal meningiomas is extrapolated from their intracranial counterparts, even though they are considered separate entities. This review aimed to systematically summarize studies covering different aspects of spinal meningiomas and their management. Methods: Databases were searched for all studies concerning spinal meningiomas dating from 2000 and onwards. When possible, a meta-analysis was performed. Results: Neurological outcomes of surgery were consistently favorable across studies, with a complication rate of 7.9% and 78.9% of the patients demonstrating good postoperative neurological function (McCormick score 1–2). The most relevant predictors of unfavorable outcomes were poor preoperative status, longer time from diagnosis to surgery, and surgery of recurrent tumors. The recurrence rate after surgery was estimated at 6%. Meta-analysis and/or survival analysis revealed that higher WHO grade (*p* < 0.001), higher Simpson grade (*p* < 0.001), ventral tumor location (*p* = 0.02), and male sex (*p* = 0.014) were all associated with higher odds of recurrence. However, the meta-analysis did not show any difference between Simpson grade 1 and grade 2 with respect to the odds of recurrence (*p* = 0.94). Surgery provided immediate and durable health-related quality-of-life improvement, as well as a high frequency of return to work. Conclusion: Spinal meningioma surgery is a relatively safe procedure with a low risk of tumor recurrence and high likelihood of favorable postoperative outcomes.

## 1. Introduction

In the first part of this systematic review, the baseline patient and tumor characteristics were studied. The topics investigated included epidemiology, tumor biology, WHO grade distribution, tumor location, presenting symptoms, and radiological diagnostics. In this second part, treatment options and outcomes were assessed by analyzing postoperative neurological status, recurrence risk, health-related quality of life, and frequency of return to work.

It is well-established that surgery is the preferred treatment of choice for spinal meningiomas [1], as tumor removal usually relieves symptoms with little risk for complications or recurrence and a high frequency of postoperative improvement [2,3,4]. Cerebrospinal fluid leak, transitory or permanent neurological impairments, and infections are the most commonly reported complications, while pulmonary embolisms were behind most of the fatalities [2,3,5,6,7]. The Simpson grading scheme is the one most commonly used in meningioma surgery to describe the extent of tumor resection and to estimate the likelihood of tumor recurrence. It is still uncertain whether removal of the dura (Simpson grade 1) prevents recurrence [2,8,9,10,11]. Tumor recurrence may occur many years after surgery. Heon Kim et al. calculated a mean clinical progression-free survival of 17 years for patients who underwent Simpson grade 2 resection, in which the dural attachment is left but coagulated [11]. Data on the health-related quality of life in patients undergoing spinal meningioma surgery is scarce, with only a few studies having addressed this issue [12,13,14]. This second part of our systematic review aims to provide a comprehensive overview of current knowledge on the treatment and outcomes of spinal meningiomas by summarizing, pooling, and, where possible, meta-analyzing the published data. This review will follow the outline proposed in the previously published protocol [15].

## 2. Materials and Methods

This systematic review was performed in accordance with the Preferred Reporting Items for Systematic Reviews and Meta-Analyses (PRISMA) guidelines [16]; the related 2020-PRISMA checklist is provided as a supplementary material (Supplementary File S1). The review protocol was registered within the International Prospective Register of Systematic Reviews (PROSPERO) (Registration ID: CRD42022330809 and Date of registration: 17 May 2022). The record was consistently updated in the event of any major change to the design of the work and the study protocol has previously been published [15]. More details regarding the methodology are stated in Part 1 of this review.

### 2.1. Eligibility Criteria

In brief, the inclusion criteria for this systematic review were peer-reviewed studies with at least ten patients, published after 2000, in the English language, covering the field of spinal meningiomas (Table 1). A control group was not deemed necessary for inclusion. Case reports, reviews, editorials, letters, and conference abstracts were excluded.

### 2.2. Databases, Search Strategy, and Study Selection

**Figure 1:**
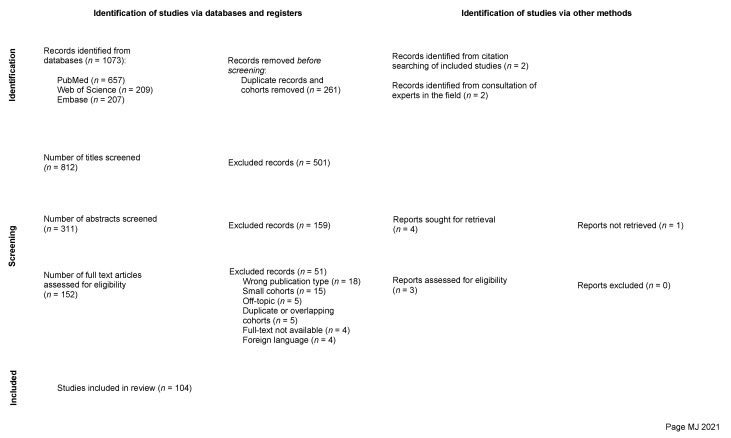
PRISMA flow chart illustrating the study selection process [16].

An electronic database search was performed in PubMed, Embase, and Web of Science. The search was based on the keywords “spinal” and “meningioma” (Supplementary File S2). The selection process was previously described in Part 1 and is illustrated in the PRISMA flowchart presented (Figure 1) [16], and the final selection of studies included in this review is provided separately (Supplementary File S3, Table S1).

### 2.3. Data Extraction

Data from selected records were collected using a predefined extraction template, including (1) general publication information, (2) patient characteristics and epidemiology, (3) intervention characteristics, (4) study characteristics, and (5) outcomes.

### 2.4. Individual Evidence Level and Risk of Bias Assessment

To rate the research design quality, the studies were grouped according to evidence level, adhering to the modified Oxford Center for Evidence-Based Medicine (OCEBM) system [17]. Most studies provided level II evidence. To evaluate the risk of bias (ROB) among the included studies, the Newcastle–Ottawa Scale (NOS) was used [18]. As most studies were single-armed, the NOS was modified to exclude modules intended for two-arm studies with comparator or control groups. Consequently, the scale allowed for a maximum of six points (instead of nine) to be allocated for each of the studies. Most studies were found to have a low overall risk of bias. An individual quality score (IQS) was then calculated for each of the studies based on both OCEBM and NOS grades. The detailed assessments are provided in the supplementary material (Supplementary File S3, Table S2).

### 2.5. Data Synthesis, Analysis, and Statistics

For a limited number of research questions, sufficient homogenous data was available to allow a meta-analysis. When the heterogeneity between studies, including methodological and design differences, prevented a meta-analysis, a pooled analysis or a qualitative synthesis was performed according to Cochrane recommendations [19]. The pooling was performed by merging the data from the included studies to obtain pooled values weighted in relation to the sample sizes of the corresponding studies. Furthermore, a thorough qualitative synthesis relied on the presentations of associations established in each study, with the corresponding significance levels, without processing or acting upon the data themselves in any way. Instead, when limited information was provided, narrative review was performed. For research questions where a meta-analysis was performed, funnel plots and Egger tests were used to assess publication bias. Moreover, the random effect model was used when the tests of heterogeneity indicated high heterogeneity among studies (*I*^2^ > 30%). In cases where heterogeneity was deemed low, the fixed-effects model was used as well. When possible, sensitivity analysis was performed by disregarding studies with less than five years of follow-up time. All statistical analyses were performed using the R software [20].

### 2.6. Quality of the Pooled Body of Evidence

Upon thorough qualitative synthesis or meta-analysis of data, regarding neurological outcomes and recurrence, the Grading of Recommendations, Assessment, Development, and Evaluation (GRADE) approach was employed to rate the body of evidence supporting the study’s key findings, assessing their strength or certainty level [21]. Details on the used technique are described in the previously published study protocol [15]. For sections where this approach was used, GRADE evidence or summary of findings tables, assembled using the GRADEpro Guideline Development Tool were provided [22].

## 3. Results and Discussion

### 3.1. Spinal Meningioma Surgery

Sixty-seven studies, including 3672 tumors, were reported on spinal meningioma surgery (Supplementary File S3, Table S9). The surgical approach was described in most studies, and the posterior midline approach was by far the most frequently used method. The posterolateral approach was the most frequently described alternative, followed by the far-lateral (typically used for upper cervical tumors). An anterior approach was applied in two cases in two different studies. One of the cases—an upper cervical meningioma—was operated transorally. The use of the anterior approach for the operation of spinal meningiomas was otherwise reported in several short case series, and not included in this review.

Laminectomy was the most common method to access the spinal canal. To a lesser extent, hemilaminectomies, laminotomies, laminoplasties, costotransversectomies, and pedicle excisions were also performed. Facetectomies were sometimes used to supplement other techniques. In one case only, a corpectomy was performed to access the tumor following an anterior approach. However, the fact that publications with less than ten patients were excluded may bias these data away from less commonly performed approaches that are more likely to be published in smaller series. The result would be an overestimation of the relative predominance of the more standard techniques.

Regarding the extent of tumor resection, the Simpson grading scheme was the most popular, with 51 studies reporting its use. The Saito method, a recently developed resection technique, was reported in four studies. Less common resection grading schemes tended to be arbitrary, inconsistent, and non-standardized across studies. These were used in eight studies in total and included different logical combinations of the following terms: “gross total”, “total”, “subtotal”, “complete”, and “incomplete” resections.

**Figure 2:**
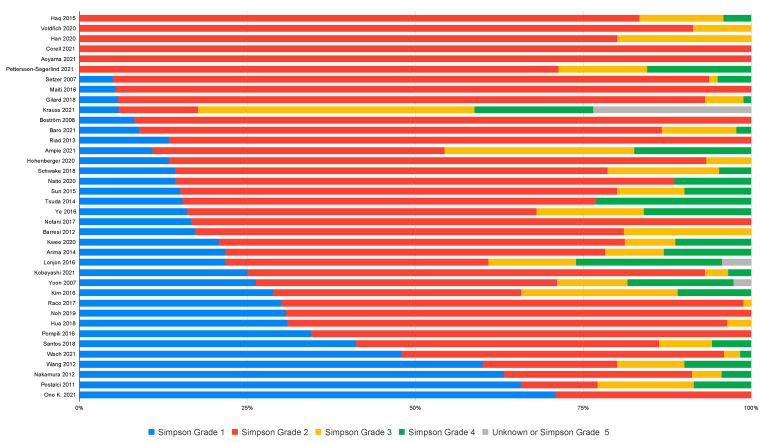
Distribution of Simpson grades as reported by the studies.

Of 51 studies employing the Simpson grade scale, 38 reported the distribution between Simpson grades. These 38 studies collectively presented data on 2327 spinal meningiomas. Simpson grades 1, 2, 3, 4, or 5 were achieved in 20.6%, 66.7%, 7.4%, 5.0%, and 0.04%, respectively, revealing a clear predominance for grade 2 resections (Figure 2).

Twelve studies provided information about the duration of surgery (Supplementary File S3, Table S9). The duration of surgery ranged from 119 to 233 min. Two of the studies associated longer surgery durations and greater blood loss to calcified tumors [23,24]. Two other studies compared duration of surgery between posterior unilateral approaches with hemilaminectomy and the traditional posterior approach with laminectomy. In the first study [25], Iacoangeli et al. chose to contrast a minimally invasive approach with the traditional one by using the Saito method along with hemilaminectomy (group 1) and a Simpson grade 1 or 2 resection strategy along with laminectomy (group 2). The average duration of surgery was reduced when performing the less invasive approach. In fact, the surgeries in the hemilaminectomy group lasted 145 min on average, compared to 171 min for the laminectomy group. The authors also found that the hemilaminectomy group had less associated bleeding during surgery [25]. Onken et al. compared duration of surgery for anteriorly versus posteriorly located tumors, accessed through either hemilaminectomy or laminectomy. For the laminectomy approach, the duration of surgery was 224 min for anterior and 148 min for posterior tumors. For the hemilaminectomy approach, the duration of surgery was 136 min for anterior and 131 min for posterior tumors [26].

Minimally invasive surgery (MIS) aims to minimize tissue trauma during surgery. In the context of spinal meningiomas, MIS is poorly defined but commonly related to the preservation of the structural integrity of the spinal column. Performing a hemilaminectomy rather than a laminectomy was often preferred by advocates of MIS [23,25,26,27,28,29,30,31]. In some cases, the length of the skin incision defined the degree of invasiveness [32]. In others, dural splitting and microsurgical resection of the inner dura while preserving the outer layer (Saito method) [25,33,34], or simply coagulation of the tumoral dura extensions (Simpson grade 2) [31] rather than complete and radical resection (Simpson grade 1) was enough to consider the approach minimally invasive. In contrast, some authors argue that surgeons should always seek approaches they are comfortable in performing [28]. However, anatomical and tumor-specific aspects such as anterior attachment or extensive tumor calcification may prompt alternative approaches [23,32,34,35,36].

Nonetheless, we believe that safe surgery and protection of the spinal cord, rather than prioritizing MIS approaches, must remain the surgeon’s main concern.

### 3.2. Intraoperative Neurophysiological Monitoring (IONM)

The use of intraoperative neurophysiological monitoring for spinal meningioma surgery was addressed in 32 studies. One study reported that the technology was not used due to limited availability, and another did not present any data on the subject, even though the authors advocated its use. In the 30 studies where the technology was employed, the frequency of use varied between authors and institutions. Eighteen studies, including a pooled total of 919 cases, reported the use of IONM during all surgeries, while the rest reported frequencies between 3.2% and 78.7%. Although the use of IONM was mentioned, the exact numbers were not presented in five of the studies.

The monitoring modality was disclosed in most of the studies (*n* = 19), with somatosensory (SEP) and motor-evoked potentials (MEP) being the most common. Only a minority of studies (*n* = 6) reported the use of a single modality, while most studies (*n* = 13) employed either two or three modalities (Supplementary File S3, Table S10).

Surgical outcomes relative to the use of IONM were reported in four studies [3,37,38,39], three of which failed to identify any significant effects [37,38,39]. One study, however, demonstrated significantly better outcomes for surgeries performed after 2009 when IONM was introduced at the authors’ institution [3].

Although advocated by many [8,23,27,39,40,41], the evidence for the use of IONM in the context of spinal meningioma surgery is lacking. The available evidence originates from a small number of studies, all pointing towards minimal to no benefit associated with its use [37,38,39,40,41]. Moreover, only one of these studies adopted a case–control design to compare surgical outcomes with vs. without IONM. This study also included spinal nerve sheath tumors in the analysis [37]. After our inclusion period, another cohort study addressing the role of IONM in spinal meningioma surgery, by comparing patients operated with and without IONM, was published. No significant improvements were detected in patients operated with IONM. The authors recommended the use of IONM only in complex cases [42]. Nonetheless, randomized controlled trials are required to explore the true effect of this methodology on outcomes in spinal meningioma surgery.

### 3.3. Operative Complications

#### 3.3.1. Section on the Risk and Severity of Postoperative Complications

In total, 49 studies on 16,751 surgically treated patients disclosed information about perioperative complications. The adjusted complication rate for the whole cohort was 7.4% with 1240 perioperative complications of different severities. At the level of the individual studies, the reported complication rate fluctuated between 0% and 50% with a mean and median of 12.5% and 9.8%, respectively.

**Figure 3:**
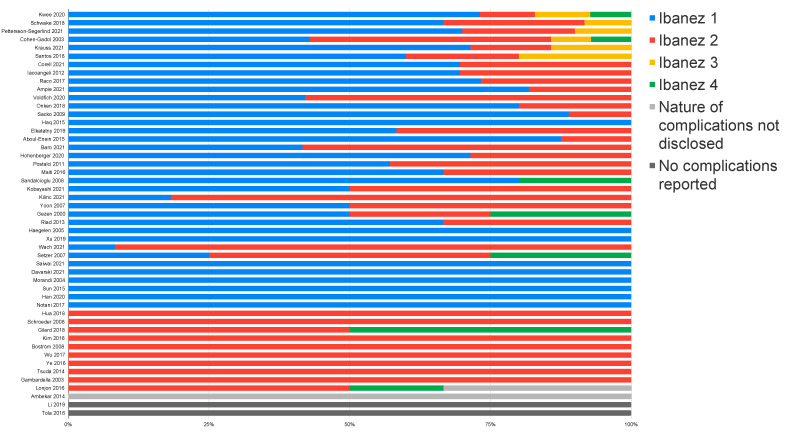
Distribution of perioperative complications according to the Ibanez grading scheme.

There were 893 complications that could not be classified according to the Ibanez grading scale due to a lack of information regarding the nature of the complications. Of these, 891 originated from a single nationwide study [43]. Of the remaining 347 complications, Ibanez grade 1 was the most frequently reported in 202 cases (58.2%). Ibanez grade 2 complications were reported in 126 cases (36.3%), while the most severe Ibanez grades 3 and 4 were uncommon, with nine (2.6%) and ten (2.9%) cases, respectively (Figure 3).

For each study, complications were ranked by order of frequency (Supplementary File S3, Table S11). The most frequently reported surgical complication was CSF leak, classified as either Ibanez grade 1 or 2 depending on the management. In cases where information on management was lacking, the CSF leak was classified as Ibanez grade 2. Second only to CSF leak, a wide range of complications were reported, such as wound infections or revisions, new neurological deficits, surgical site pain, or hematomas. Severe complications such as death, coma, iatrogenic spinal cord injury, instability that required fixation, and thromboembolism were relatively rare. Thromboembolism was the most common of the severe complications and the most common cause of perioperative death in patients surgically treated for spinal meningiomas.

#### 3.3.2. Factors Influencing the Complication Rate

Three studies reported complication rates in elderly (>70 years old) patients undergoing surgery for spinal meningiomas [25,44,45]. Based on 197 cases, a cumulative complication rate of 16.8% was found. This exceeds the rate of 7.4% calculated from all studies in this review where the information was available. Schwake et al. stated that most of the 12 complications in their cohort of 84 operated patients occurred in elderly patients [29]. These results are in conflict with two other studies that could not associate age to a higher complication rate [2,43], including the largest study in this section with 13,698 patients [43].

The combination of three studies [34,35,46] with 38 ventral tumors yielded a pooled complication rate of 26.3%, with cervical tumors accounting for most of the complications. Patients with ventral spinal meningiomas may hence carry a higher risk of complications [41]. One study found significant ties between CSF fistula formation and ventral tumor location, with an odds ratio (OR) estimated at 10.5 [47].

Other factors significantly associated with an increased risk of perioperative complications were obesity [47,48] (OR: 3.2 in one study [49]), surgery for recurrent tumors [48,49,50], surgeon inexperience [48], and tumor calcification [30]. Additionally, longer operations were associated with an increased risk of postoperative CSF leak [48]. The potential effect of WHO grade on the complication rate was rarely mentioned. In the five studies on spinal meningiomas with higher WHO grades [51,52,53,54,55], ten complications were detected in the 94 patients included, amounting to a complication rate of 10.6%. Less invasive procedures may be associated with lower complication rates. Three studies compared Simpson grade 2 resections with the Saito method in terms of the risk of postoperative CSF leak [25,33,34]. In total, 5 of the 72 patients in the Simpson grade 2 group (7%) and 2 of the 46 in the Saito group (4%) developed CSF leaks. In one study, CSF leaks could neither be associated to Simpson grade (1 vs. 2), nor to the number of spinal segments involved [47]. Two studies found no difference in the complication rates of unilateral and bilateral laminectomies [25,26].

In summary, spinal meningioma surgery is a relatively safe procedure with a complication risk of 7.4%. Although most of these complications are mild and resolve conservatively (Ibanez 1, 58.2%), there are also more severe complications requiring invasive treatment (Ibanez 2, 36.3%) or leading to severe impairment and even death (Ibanez 3 and 4, 5.5%). Ibanez grade 1 complications, such as postoperative urinary tract infections or diarrhea, may be underreported due to their relative insignificance. In total, severe complications characterized as Ibanez 3 or 4 occurred in 0.11% of all 16,751 spinal meningioma surgeries regarded in this section. Although rare, thrombosis prevention strategies in target patients could help reduce the risk of Ibanez 4 complications, which mainly consisted of thromboembolic events [36]. Due to the lack of studies analyzing the predictors of perioperative complications in spinal meningioma surgery, solid risk factors for the development of these events have yet to be established.

### 3.4. Neurological Outcomes

#### 3.4.1. Neurologic Impairment Scales Used

Fifty-eight studies evaluated functional outcomes (Supplementary File S3, Table S12). Different scales or grading schemes were used to determine neurological function, performance, and pain levels. In the reviewed literature, the most common grading scheme for assessment of neurological function was the McCormick scale (MCS), which was employed in 28 different studies. Of these, 18 studies utilized the modified version (mMCS), while 10 reported the use of the original scale as described by McCormick et al. in 1990 [56]. The Karnofsky performance score (KPS) [57] and Frankel grade [58] were second and third, with nine and eight studies reporting their use, respectively. Other scales, such as the American Society of Anesthesiologists (ASA) (*n* = 5), the Nurick (*n* = 5), the Medical Research Council (MRC) (*n* = 3), the Japanese Orthopedic Association (JOA) (*n* = 2), the Klekamp and Samii (*n* = 1), the Solero (*n* = 1), the Levy (*n* = 1), and the Eastern Cooperative Oncology Group (ECOG) (*n* = 1), were less popular. Furthermore, the visual analogue scale (VAS) for pain was utilized in four studies. Several studies used more than one of these scales to report patient outcomes.

#### 3.4.2. Postoperative Neurological Outcome

Based on the studies reporting neurological function according to the McCormick scale (MCS), the data were pooled and dichotomized to good (MCS 1–2) or poor (MCS ≥ 3). Regarding the preoperative function, 24 studies reported data on 1633 patients, and of these, 876 (53.6%) had a good MCS, while the remainder (46.4%) had a poor MCS. In the 22 studies presenting the postoperative status of 1432 patients, 1130 (78.9%) had good MCS while the remainder (21.1%) had poor MCS. Similar results could be deduced using the Frankel scale for the eight studies where this information was available.

The rate of postoperative improvement, stabilization, or worsening of neurological status was calculated from 42 different studies. Most patients either improved (65.2%) or remained unchanged (28.8%). It is worth noting that patients with MCS 1 cannot improve any further and are likely a major contributor to the proportion of patients remaining postoperatively unchanged. The incidence of postoperative worsening was insignificant (6.0%). At the level of individual studies, the incidence of postoperative worsening or deterioration rate ranged from 0% to 15.2% (median: 4.1%, mean: 5.3%).

In one study on stereotactic radiosurgery, post-intervention outcomes were reported [59]: the majority remained stable (94.1%), 5.9% (*n* = 1) improved, and no patients deteriorated. Finally, a comparison of pre- and postoperative symptoms was performed in 18 studies. Symptom-specific postoperative improvement was reported for the absolute majority of patients in most studies. Nine studies concluded that bladder or bowel dysfunction improved to a lesser degree than other symptoms. This finding was more pronounced in the elderly according to one of the studies [60]. In fact, resolution of bladder or bowel dysfunction may occur in only half of patients [60].

#### 3.4.3. Outcome Predictors

**Figure 4:**
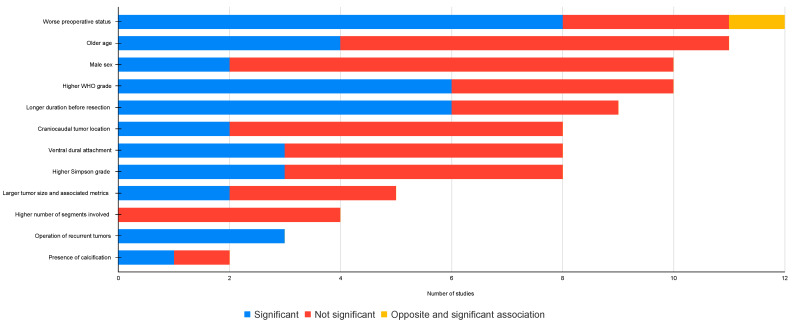
Statistical significance status of different neurological prognostic markers.

Twenty-one studies reported different predictors of unfavorable outcomes, defined as neurological deterioration or poor neurological function (Supplementary File S3, Table S13). A qualitative synthesis was performed, including the number of studies addressing each of the predictors, the level of bias, and the level of significance (Figure 4). All 21 studies in this section had a low level of bias.

Preoperative function: the most commonly cited predictor was the preoperative neurological status of the patient, studied in 12 articles. Eight studies found a worse preoperative neurological status to be associated with unfavorable outcomes, and one study found the opposite to be true [61]. The remainder of the studies could not reach statistical significance (Supplementary File S3, Table S13).

Age: The association between age and unfavorable outcomes was reported in 11 studies. Older age was significantly correlated to unfavorable outcomes in four studies. Two studies on 95 elderly patients (>68 years) revealed a pooled postoperative improvement frequency of 96.8% [25,44], contrary to the belief that older patients are at higher risk of postoperative deterioration (Supplementary File S3, Table S13).

Sex: the effect of sex on poor postoperative scores or neurological deterioration was reported in ten studies. In all studies, male sex was hypothesized to be a predictor of unfavorable outcomes. However, only two of the studies could confirm this (Supplementary File S3, Table S13).

WHO grade: a higher WHO grade was associated to unfavorable outcomes in six of ten studies (Supplementary File S3, Table S13).

Time to surgery: a longer duration of symptoms or longer time to surgery was associated with unfavorable outcomes in six of eight studies (Supplementary File S3, Table S13).

Craniocaudal tumor location: six studies found no correlation between the craniocaudal location of the tumor and an unfavorable outcome (Supplementary File S3, Table S13).

Axial tumor location: three of eight studies found an association between a ventral tumor location and unfavorable outcomes (Supplementary File S3, Table S13).

Simpson grade: a higher Simpson grade was associated with worse outcomes in three of eight studies (Supplementary File S3, Table S13).

Tumor size and spinal cord compression: a larger tumor size was associated with worse outcomes in two out of five studies (Supplementary File S3, Table S13).

Number of segments involved: none of the four studies covering this topic could find a statistically significant correlation (Supplementary File S3, Table S13).

Recurrent tumor: Excision of a recurrent tumor was associated with significantly worse outcomes in all three studies where this factor was analyzed (Supplementary File S3, Table S13). These findings were primarily attributed to fibrosis resulting from the index surgical intervention.

Tumor calcification: only one of two studies found a significant correlation between extensive tumor calcification and unfavorable outcomes.

Other correlations (Supplementary File S3, Table S13): other less frequently studied predictors of unfavorable postoperative outcomes were, race (not significant), NF2 diagnosis (not significant), or presence of comorbidities (significant).

Overall, 78.9% of the patients had a good postoperative outcome on the MCS, and only 6% worsened. Unfavorable outcomes, defined as either postoperative deterioration or poor postoperative neurological function, are relevant from both quality of life and socioeconomical standpoints, as they may cause longer hospital stays [48] and a larger economic burden on patients and the health care system. Predictors of unfavorable outcomes were studied in several studies. Unfortunately, the associated significance levels were often contradictory.

A summary of all potential predictors, supported by at least one study with statistically significant findings, was generated using the GRADE approach. This approach considers the study design, risk of bias, inconsistencies, imprecisions, indirectness, and magnitude of the associations [15] to determine the level of evidence for each predictor (Table 2).

The most relevant predictors of unfavorable outcomes were poor preoperative status and longer time to surgery, both supported by evidence of moderate certainty, and surgery for recurrent tumors, supported by evidence of high certainty. The effect of age, sex, number of segments, craniocaudal and axial tumor locations, WHO and Simpson grades and tumor calcification on outcome was supported by either low or very low certainty levels.

### 3.5. Tumor Recurrence

#### 3.5.1. Overall Recurrence Rate

Sixty studies reported on recurrence of spinal meningiomas after index surgery. Of these studies, five covered higher WHO grade tumors [52,53,54,55,62], four ventral tumors [34,35,46,63], two elderly patients [44,45], and two pediatric patients [64,65]. The remaining studies (*n* = 47), including 3195 cases, were representative of the entire population of patients with spinal meningiomas. These studies reported recurrence rates ranging from 0% to 25% (median: 4.8%), while the pooled recurrence rate, which represents the 192 cases of recurrence that had occurred among the 3195 surgically treated tumors, was 6.0%.

The length of follow-up was reported in all studies. The average follow-up time ranged from 14 months to over ten years (median: 53.5 months), with an overall weighted average of roughly five years (62.9 months). Approximately 95% of the studies had follow-up times beyond the two-year mark, while only 40% extended beyond five years. Only five studies had a follow-up time longer than ten years [5,8,9,11,33]. A positive correlation was identified between recurrence rates and length of follow-up (Pearson correlation coefficient: R = 0.36, *p* = 0.021, Supplementary File S3, Figure S1).

The time to recurrence was reported in 40 studies. The pooled mean time to recurrence was estimated at almost 5 years (59.8 months). Thus, the average time to recurrence is well beyond the median follow-up time in all studies, but comparable to the weighted average follow-up time (62.9 months), suggesting that the follow-up time in at least half of the studies may have been insufficient to identify most cases of recurrence. In the collected material, 27 of 144 recurrences with a known time to recurrence (18.8%) occurred after 96 months (8 years) of follow-up. This may have led to a severe underestimation of the true recurrence rate of surgically treated spinal meningiomas.

Potential risk factors for recurrence were statistically analyzed in 19 studies (Supplementary File S3, Table S14).

#### 3.5.2. Recurrence and Patient Characteristics

The two pediatric studies included in this section reported recurrence rates of 28.6% and 70.0%, resulting in a pooled recurrence rate of 45.8% [44,45]. In contrast, two studies focusing on the elderly reported negligible recurrence rates of 0% and 0.98%, resulting in a pooled recurrence rate of 0.8% [64,65]. The average follow-up times were similar at around 40 and 70 months in the pediatric cohorts, and at 50 and 60 months in the cohorts of the elderly. Thus, tumor recurrence was considerably more frequent in pediatric cohorts. Moreover, four studies revealed a significant negative correlation between patient age and the risk of recurrence, while seven studies could not demonstrate any significant relation between the two.

**Figure 5:**
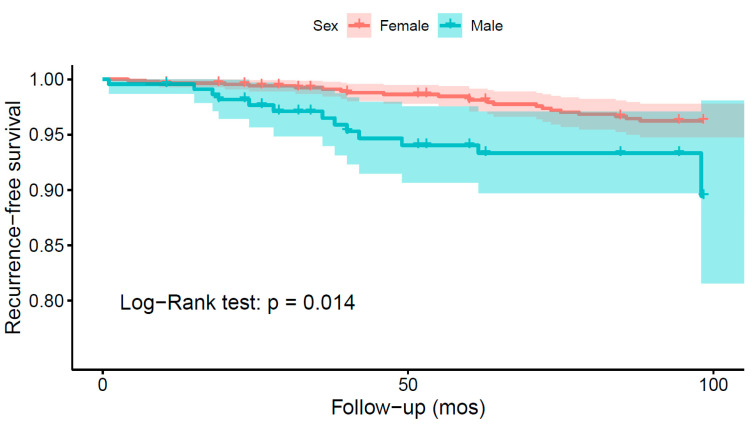
Kaplan–Meier survival analysis comparing the recurrence-free survival of spinal meningioma patients depending on sex.

Six studies analyzed sex as a risk factor for recurrence. While four of the studies associated the male sex to a higher risk of recurrence, two studies could not find a statistically significant association (Supplementary File S3, Table S14). A Kaplan–Meier recurrence-free survival analysis was performed based on the data from 17 different studies on 1118 patients, revealing a shorter recurrence-free survival in males (Figure 5; log-rank test: *p* = 0.014). All studies included in the analysis had moderate to low risk of bias.

#### 3.5.3. Recurrence and WHO Grade

**Figure 6:**
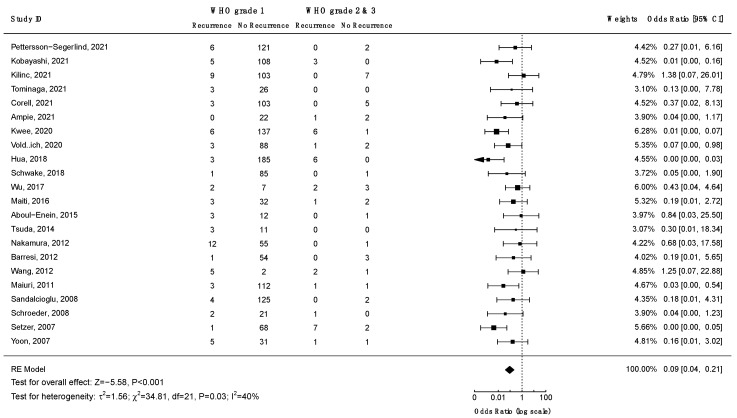
Meta-analysis of the odds ratio for recurrence of low (1) vs. high WHO grade (2 and 3) spinal meningiomas. CI = confidence interval; RE model = random effects model; Z = test for overall effect; τ^2^ = between-study variance; *x*^2^ = chi-squared test; df = degree of freedom; I^2^ = percentage of variance in a meta-analysis attributable to study heterogeneity.

**Figure 7:**
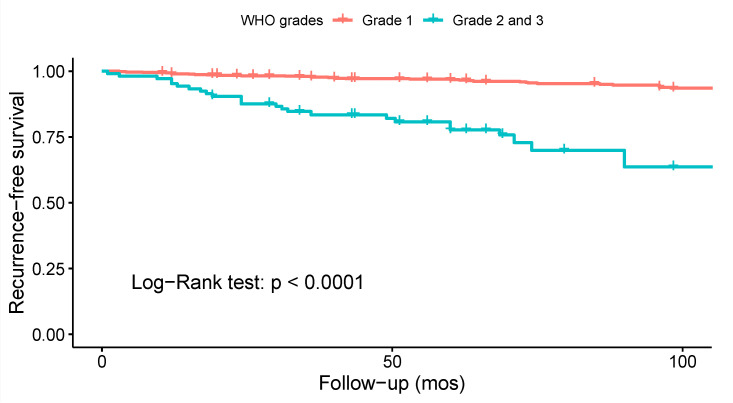
Kaplan–Meier survival analysis comparing the recurrence-free survival of patients with low (1) vs. high WHO grade (2 and 3) spinal meningiomas.

Ten studies investigated the association between WHO grade and the risk of recurrence. Only four of them reported statistically significant findings (Supplementary File S3, Table S14). A meta-analysis on the data from 22 studies (Figure 6), revealed decreased odds of recurrence for WHO grade 1 spinal meningiomas as compared to WHO grades 2 and 3: OR = 0.09 (CI 95% [0.04:0.21], *p* < 0.001). Similarly, a Kaplan–Meier recurrence-free survival analysis on data from 26 studies and 1070 patients revealed significant results favoring WHO grade 1 tumors (log-rank test: *p* < 0.001; Figure 7). According to the Funnel plot and Egger’s test, publication bias was negligible (Supplementary File S3, Figure S2; *p* = 0.3). All studies included in this analysis had low risk of bias. A sensitivity analysis including only studies with an average follow-up duration of at least five years had no impact on the results.

#### 3.5.4. Recurrence and Axial Tumor Location

**Figure 8:**
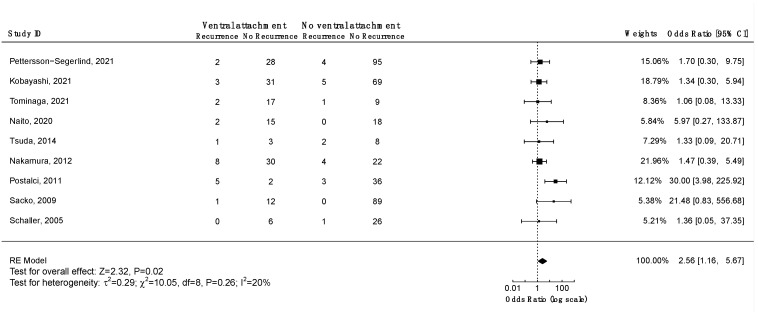
Meta-analysis of the recurrence odds ratio of ventral vs. non-ventral spinal meningiomas. CI = confidence interval; RE model = random effects model; Z = test for overall effect; τ^2^ = between-study variance; *x*^2^ = chi-squared test; df = degree of freedom; I^2^ = percentage of variance in a meta-analysis attributable to study heterogeneity.

Four studies investigated the association between ventral tumor location and risk of recurrence. Only one study associated ventral tumor location with a higher risk of recurrence (Supplementary File S3, Table S14). A meta-analysis including nine studies on 560 patients was performed (Figure 8). A significant positive association was found between ventral tumor location and risk of recurrence (OR = 2.56, CI 95% [1.16:5.67], *p* = 0.02). According to the Funnel plot and Egger’s test, publication bias was negligible (Supplementary File S3, Figure S3; *p* = 0.3). Similar results were obtained using the fixed-effect model. A sensitivity analysis including only studies with an average follow-up duration of at least five years had no impact on the results. All studies had a low risk of bias.

#### 3.5.5. Recurrence and Extent of Resection

**Figure 9:**
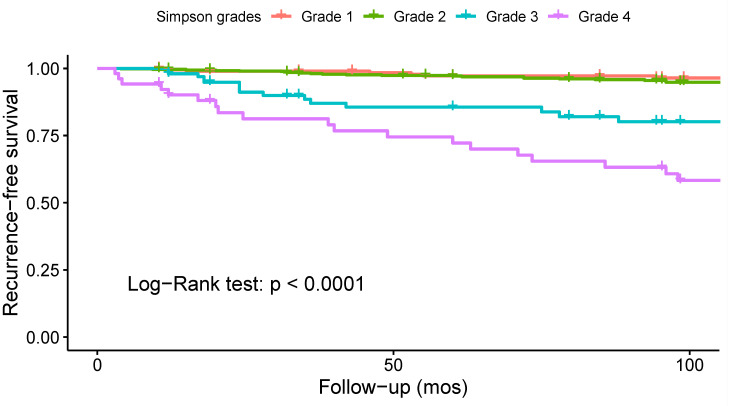
Kaplan–Meier survival analysis comparing the recurrence-free survival of patients operated with the different Simpson resection grades.

**Figure 10:**
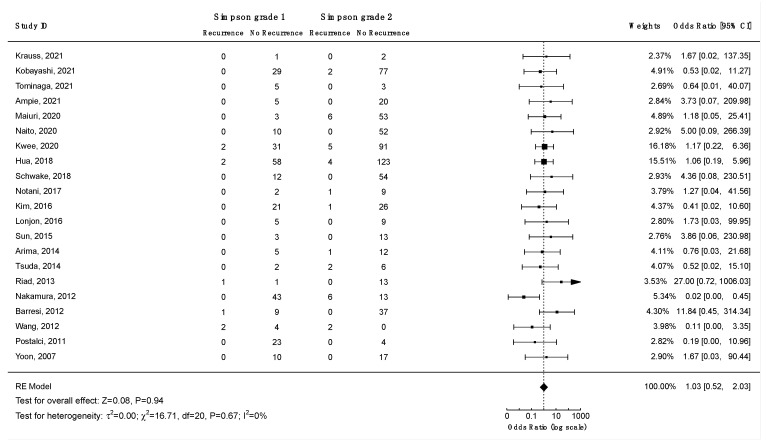
Meta-analysis of the recurrence odds ratio for spinal meningiomas after Simpson grade 1 vs. grade 2 resections. CI = confidence interval; RE model = random effects model; Z = test for overall effect; τ^2^ = between-study variance; *x*^2^ = chi-squared test; df = degree of freedom; I^2^ = percentage of variance in a meta-analysis attributable to study heterogeneity.

**Figure 11:**
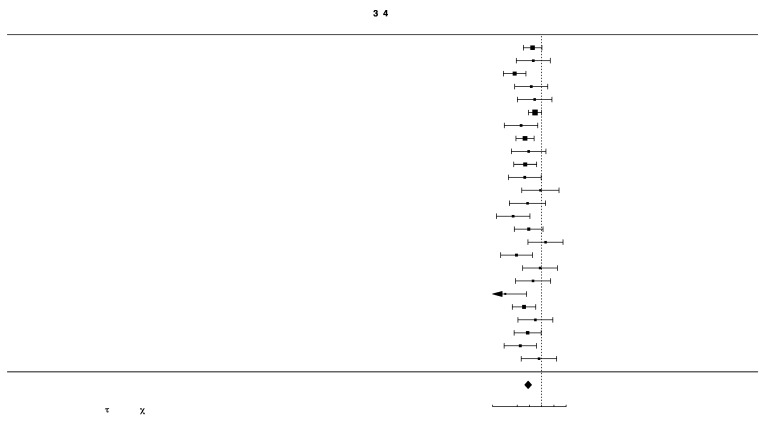
Meta-analysis of the recurrence odds ratio for spinal meningiomas after Simpson grade 1–2 vs. grade 3–5 resections. CI = confidence interval; RE model = random effects model; Z = test for overall effect; τ^2^ = between-study variance; *x*^2^ = chi-squared test; df = degree of freedom; I^2^ = percentage of variance in a meta-analysis attributable to study heterogeneity.

Thirteen studies investigated the association between the Simpson grade and the risk of recurrence, and eight of them found a statistically significant correlation (Supplementary File S3, Table S14). To analyze the impact of Simpson grade on the risk of recurrence, a Kaplan–Meier curve was plotted using data from 16 studies on 950 patients (Figure 9). The analysis revealed an increased frequency of recurrences with higher Simpson grades over time (log-rank test: *p* < 0.001). To further investigate these findings, two separate meta-analyses were performed to assess the differences in odds of recurrence for Simpson grades 1 vs. 2 (Figure 10) and 1 or 2 vs. 3, 4, or 5 (Figure 11). The difference in odds of recurrence for Simpson grade 1 compared to Simpson grade 2 was insignificant (21 studies, 959 patients: OR = 1.03; CI 95% [0.52:2.03]; *p* = 0.94). The second meta-analysis comparing Simpson grades 1 or 2 versus Simpson grades 3, 4, or 5 was indicative of a higher risk of recurrence for the latter (25 studies, 1641 patients: OR = 0.08; CI 95% [0.04:0.15]; *p* < 0.001). Funnel plots (Supplementary File S3, Figures S4 and S5) and Egger’s tests were employed to assess the risk of publication bias. In both cases, publication bias was negligible (*p* = 0.6 and *p* = 0.8, respectively). Similar results were obtained using the fixed-effect models. A sensitivity analysis including only studies with an average follow-up duration of at least five years had minimal impact on the results. All studies included in this analysis exhibited a moderate to low level of bias.

Only three studies reported on the dura preservation method (Saito method) [5,33,34], which precluded a meta-analysis of data. However, pooling of the data revealed comparable recurrence rates for the dura preservation method and Simpson grade 2 resection, where the dural attachments are left but coagulated (8.3% vs. 10%).

#### 3.5.6. Other Risk Factors for Recurrence

Craniocaudal tumor location: The relation between craniocaudal location and tumor recurrence risk was studied in seven studies with a low risk of bias. Only one study found an association (Supplementary File S3, Table S14).

MIB1-index: The proliferation index was analyzed as a risk factor for recurrence in three studies with low risk of bias. Two studies found an association between a higher MIB-1 index and a higher risk for recurrence (Supplementary File S3, Table S14).

Number of segments involved: The relation between number of segments involved and tumor recurrence was studied in four studies with a low risk of bias. All but one study found no association between a higher number of segments involved and a higher risk for recurrence (Supplementary File S3, Table S14).

Tumor calcification: The association between tumor calcification and the risk of tumor recurrence was assessed in two studies with a low risk of bias. Only one of the studies could verify the association (Supplementary File S3, Table S14).

Surgical expertise: Kilinc et al. studied the impact of the operating surgeon’s level of experience on the risk of recurrence but could not find any statistically significant differences [48]. The risk of bias associated with this study was low.

In summary, the overall recurrence rate after spinal meningioma surgery is close to 6%, with an average of 5 years from index surgery to recurrence. Half of the studies had follow-up times shorter than the average time to recurrence and a large number of tumors recurred after eight to ten years. Hence, the true incidence of recurrence is likely to be underestimated, suggesting that radiological follow-ups should be extended, especially for younger patients.

Age and sex influenced the risk of recurrence. Higher recurrence rates were observed in younger patients compared to the general population of patients with spinal meningiomas and may have been due to an overrepresentation of NF2 cases, as well as higher WHO grades in this cohort. On the other hand, older patients (>70 years) demonstrated lower recurrence rates. The reduced life expectancy in this segment of the population may certainly be part of the explanation [2].

Only a fraction of the studies analyzed risk factors associated with recurrence, and the results were often contradictory. Pooling of the data, meta-analysis, and survival analysis for further validation could only be performed for a limited number of research questions due to the lack of available data.

Kaplan–Meier recurrence-free survival analyses were performed to visualize outcome differences for sex and the WHO and Simpson grades. The analyses revealed significantly higher recurrence rates in males (*p* = 0.014), for WHO grade 2 and 3 tumors (*p* < 0.001), and for tumors resected using Simpson grades 3, 4, or 5 (*p* < 0.001). Meta-analyses were performed to assess the differences in the odds of recurrence depending on Simpson and WHO grades as well as the axial tumor location. The GRADE approach was employed to assess the certainty level of the evidence (Table 3). Meta-analyses corroborated that higher WHO grades (*p* < 0.001) and higher Simpson grades (*p* < 0.001) increase the odds of recurrence. In both cases, the certainty level of the evidence was high. Furthermore, a meta-analysis of the odds of recurrence relative to the axial tumor location revealed a significant ventral predilection among recurrent cases (*p* = 0.02). The certainty of the evidence was moderate.

To address one of the most debated questions in the field, a meta-analysis was performed comparing the recurrence rates between Simpson grade 1 and grade 2 resections. No significant differences were found between Simpson grades 1 and 2 in preventing recurrence (*p* = 0.94). Hence, coagulating the tumor attachment to preserve the integrity of the dura, rather than resecting it to achieve Simpson grade 1, may be viewed as a safe and effective approach in the treatment of spinal meningiomas.

### 3.6. Quality-of-Life after Spinal Meningioma Surgery

Three studies reported on the postoperative health-related quality of life for spinal meningioma patients. Two of the studies included mixed intradural extramedullary spinal tumors [12,66], and one included only patients with spinal meningioma [13].

In the study by Vierech et al., 44 patients, 14 with spinal meningiomas, were followed for 12 months postoperatively using standardized questionnaires. The authors reported that the disability index; the ability to perform usual activities; pain scores; and discomfort, anxiety, and depression steadily improved over the postoperative follow-up period, while mobility and self-care deteriorated in the immediate postoperative period (<1 month) before subsequent improvement was seen.

In a prospective study by Newman et al., 57 patients, among them 18 spinal meningiomas, were assessed both pre- and postoperatively using standardized questionnaires including the Brief Pain Inventory (BPI) and MD Anderson Symptom Inventory (MDASI). Surgical resection resulted in statistically significant improvements in pain severity, pain interference, and overall pain experience, as well as all composite scores for the MDASI.

In one study by Pettersson–Segerlind et al. [13], the health-related quality of life was analyzed in 84 patients with spinal meningiomas, at a mean follow-up of almost 9 years (104.4 months). The results were compared to historical data from a local cross-sectional survey of a representative matched sample of the general population. No significant differences in quality of life were found between the study cohort and the matched population sample. It was also revealed that all patients who worked preoperatively had returned to work in the postoperative period, and most often within three months after surgery. Almost all patients (96%) answered that they would accept the same surgery if asked again. The authors concluded that the surgical treatment of spinal meningiomas should not be seen as a threat to long-term health-related quality of life.

Despite many studies evaluating the neurological outcome after surgery for spinal meningiomas, few studies investigate health-related quality-of-life and return to work. All data available on this topic originated from three studies, two of which considered cohorts of mixed intradural extramedullary tumors [12,66]. Only one study examined spinal meningioma patients exclusively [13]. All studies used standardized tools to assess health-related quality-of-life. Finally, all three studies concluded that surgery results in improved health-related quality-of-life measures and a high frequency of return to work.

### 3.7. Limitations

Despite including 104 studies, this systematic review has several limitations. First, not all studies were included in each section of the systematic review. This has to do with the nature of the material and the data provided by each study. Second, only a few questions could be addressed using meta-analysis due to the heterogeneity of study designs, study populations, and methodologies of the included studies. Third, the extent to which conclusions can be drawn from the findings of this review is limited by the quality of the studies included. Limitations to study designs, such as the retrospective nature of most studies, must be considered. Fourth, certain regions of the world were less represented in the reviewed body of literature, hence limiting the generalizability of the results. In fact, most of the studies reflect the situation in high-income countries, while few to none consider low- and middle-income countries. This opposes our goal to provide data representative of the global population, and instead highlights a gap in the medical literature.

## 4. Conclusions

Spinal meningioma surgery was found to be safe and effective. Most patients (78.9%) had good postoperative outcomes, and only a small minority (6%) experienced postoperative worsening. The most relevant and consistent predictors of unfavorable outcomes across studies were poor preoperative status, longer time to surgery, and surgery of recurrent tumors. Thus, early imaging of patients with symptoms of spinal cord compression in combination with effective referral systems are needed to minimize doctor delay in preoperative management.

The overall perioperative complication risk was 7.4% and most complications were mild (Ibanez type 1, 58.2% of all complications). Complications that required revision surgery (Ibanez type 2, 36.3% of all complications) predominantly included spinal fluid leakage. However, the granularity of the data did not allow for a detailed analysis of the frequency of postoperative spinal fluid leakage. Thromboembolic events were the main cause of the few severe complications (Ibanez type 3 or 4, 5.5% of all complications), highlighting the need for preventive measures in the postoperative management of risk patients. There was no evidence to suggest that IONM reduces the frequency of complications [40,41,42].

The overall rate of recurrence after spinal meningioma surgery was estimated at 6%. The average time to recurrence was five years after index surgery, suggesting the need for radiological follow-ups during many years, especially for younger patients. In the pediatric population (<18 years), relatively high recurrence rates were seen. In contrast, elderly patients (>70 years) demonstrated lower recurrence rates in the pooled analysis.

Survival analyses revealed significantly higher risks of recurrence in males (*p* = 0.014), WHO grade 2 and 3 tumors (*p* < 0.001), and tumors operated with Simpson grade 3 or higher (*p* < 0.001). Meta-analyses further validated the impact of higher WHO grades (*p* < 0.001) and higher Simpson grades (*p* < 0.001) on the odds of recurrence. Moreover, meta-analysis of the odds of recurrence relative to the tumor location in the axial plane revealed a significant ventral predilection (*p* = 0.02). Arguably, this reflects the inherent difficulties in surgically treating anteriorly located tumors. In want of more effective surgical or adjuvant treatments, anteriorly located tumors should be given special consideration during the postoperative follow up.

Of note, meta-analysis of the data did not reveal any significant difference between Simpson grades 1 and 2 with respect to the odds of tumor recurrence (*p* = 0.94). Hence, preserving the integrity of the dura via coagulation (Simpson grade 2) and avoiding aggressive resection of the dura to achieve Simpson grade 1 may be viewed as a safe and effective approach in the treatment of spinal meningiomas.

Finally, surgery of spinal meningiomas provided a durable improvement of health-related quality-of-life measures, and a high frequency of return to work.

## Figures and Tables

**Table 1:** Ibanez complication grading scheme.

Grade	Nature of the Complication	Example of Complication
1	Any medical or surgical non–life-threatening deviation from normal operative/postoperative course, requiring either non-invasive treatment or no treatment at all.	- Medical: acute urinary retention, non-infectious diarrhea - Surgical: CSF leak not requiring reoperation, seizures or CNS infections managed with appropriate treatments
2	Medical or surgical complication requiring invasive treatment, with or without general anesthesia.	- Medical: deep vein thrombosis requiring vena cava filter - Surgical: wound revision, CSF leak requiring surgical repair or drainage
3	Medical or surgical life-threatening complications requiring ICU care, with either single or multi-organ failure.	- Medical: acute myocardial infarction, lung distress, renal failure - Surgical: acute hydrocephalus requiring drainage, severe meningitis, iatrogenic ischemia
4	Any medical or surgical complication leading to operative or postoperative death.	Perioperative death as the ultimate endpoint

CSF: cerebrospinal fluid, CNS: central nervous system, ICU: intensive care unit.

**Table 2:** Narrative GRADE evidence summary table for markers of unfavorable postoperative outcomes.

Number of Studies	Certainty Assessment						Impact	Certainty	Importance
	Study Design	Risk of Bias	Inconsistency	Indirectness	Imprecision	Other Considerations			
(1) Worse preoperative status									
12	Observational studies	Not serious	Serious ^a^	Serious ^b^	Not serious	Strong association	There seems to be evidence linking worse preoperative status to unfavorable outcomes, knowing that most of the studies taking part in this analysis had low risks of bias and showed statistically significant results.	⨁⨁⨁◯ Moderate	CRITICAL
(2) Older age									
11	Observational studies	Not serious	Very serious ^c^	Not serious	Not serious	None	The body of evidence supporting older age as a marker of unfavorable outcomes is mostly relying on studies with statistically insignificant results, hence the low certainty level.	⨁⨁◯◯ Low	NOT IMPORTANT
(3) Male sex									
10	Observational studies	Not serious	Very serious ^c^	Not serious	Serious ^d^	None	The evidence suggesting male sex as an indicator of poor outcome is backed by a majority of studies showing insignificant results, which is reflected by very low certainty levels.	⨁◯◯◯ Very low	NOT IMPORTANT
(4) Higher WHO grade									
10	Observational studies	Not serious	Serious ^a^	Not serious	Serious ^d^	None	There seems to be weak evidence linking tumors with higher WHO grades to unfavorable postoperative outcomes, especially that most studies studying this association revealed insignificant results.	⨁⨁◯◯ Low	IMPORTANT
(5) Longer duration of symptoms or longer waiting time before surgery									
9	Observational studies	Not serious	Serious ^a^	Serious ^b^	Not serious	Strong association	The evidence supporting longer timespans before surgery as a marker for poor postoperative outcomes seems to be of moderate strength. Studies that supported this claim were unbiased, and most of them had initially concluded significant results.	⨁⨁⨁◯ Moderate	CRITICAL
(6) Craniocaudal tumor location									
8	Observational studies	Not serious	Very serious ^c^	Not serious	Not serious	None	The claim that spinal meningiomas of specific spinal levels may be associated to worse postoperative outcomes is based on studies revealing statistically insignificant results, hence justifying the low level of certainty towards the evidence.	⨁⨁◯◯ Low	NOT IMPORTANT
(7) Ventral attachment									
8	Observational studies	Not serious	Very serious ^c^	Not serious	Not serious	None	There is low-certainty evidence backing up the association between tumors of ventral origin and unfavorable postoperative outcomes, mainly stemming from the insignificant results found by most studies included in the synthesis.	⨁⨁◯◯ Low	NOT IMPORTANT
(8) Higher Simpson grade									
8	Observational studies	Not serious	Very serious ^c^	Not serious	Not serious	None	There is low-certainty evidence backing up the association between a higher Simpson resection grade and unfavorable postoperative outcomes, mainly stemming from the insignificant results found by most studies included in the synthesis.	⨁⨁◯◯ Low	NOT IMPORTANT
(9) Larger tumor size and spinal cord compression									
5	Observational studies	Not serious	Very serious ^c^	Very serious ^b^	Not serious	None	The evidence suggesting larger tumor sizes as an indicator of poor outcome is backed by a majority of studies showing insignificant results, which is reflected by very low certainty levels.	⨁◯◯◯ Very low	NOT IMPORTANT
(10) Surgery for recurrent tumor									
3	Observational studies	Not serious	Not serious	Not serious	Serious ^e^	Strong association	There seems to be strong evidence suggesting that the reoperation of tumors may be associated with unfavorable postoperative outcomes.	⨁⨁⨁⨁ High	CRITICAL
(11) Presence of calcification									
2	Observational studies	Not serious	Very serious ^c^	Not serious	Very serious ^d,e^	None	There seems to be poor evidence suggesting an association between tumor calcification and worse postoperative outcomes, as there were only two studies that addressed this aspect with mixed-significance results.	⨁◯◯◯ Very low	NOT IMPORTANT

^a^: Moderately conflicting significance levels across studies, ^b^: different surrogates were used throughout the studies, ^c^: severely conflicting significance levels across studies, ^d^: relatively few patients and few events were considered in the analysis, ^e^: few studies were considered in the analysis.

**Table 3:** GRADE evidence table summarizing the relevant risk factors for tumor recurrence.

Certainty Assessment								No. of Patients		Effect		Certainty	Significance and Importance
Number of Studies	Mean Follow-Up (mos)	Study Design	Risk of Bias	Inconsistency	Indirectness	Imprecision	Other Considerations	Simpson Grade 1	Simpson Grade 2	Relative (95% CI)	Absolute (95% CI)		
(1) Simpson grades 1 or 2 vs. Simpson grades 3, 4 or 5 resection of spinal meningiomas													
25	62.4	Observational studies	Not serious	Not serious	Not serious	Not serious	Very strong association	41/1326 (3.1%)	74/266 (27.8%)	OR 0.08 (0.04 to 0.15)	248 fewer per 1000 (from 263 fewer to 224 fewer)	⨁⨁⨁⨁High	SIGNIFICANT, CRITICAL
(2) WHO grade 1 vs. WHO grade 2 or 3 spinal meningiomas													
22	53.2	Observational studies	Not serious	Not serious	Not serious	Not serious	Very strong association	83/1591 (5.2%)	32/69 (46.4%)	OR 0.09 (0.04 to 0.21)	392 fewer per 1000 (from 430 fewer to 310 fewer)	⨁⨁⨁⨁High	SIGNIFICANT, CRITICAL
(3) Ventral vs. non-ventral spinal meningiomas													
9	70.9	Observational studies	Not serious	Not serious	Not serious	Not serious	Strong association	20/392 (5.1%)	24/168 (14.3%)	OR 2.56 (1.16 to 5.67)	156 more per 1000 (from 19 more to 343 more)	⨁⨁⨁◯Moderate	SIGNIFICANT, CRITICAL

CI: confidence interval; OR: odds ratio.

## Supplementary Materials

The following supporting information can be downloaded at: https://www.mdpi.com/article/10.3390/cancers14246221/s1, File S1. PRISMA checklist (part 2); File S2: Search strategy; File S3: Figures and tables: Figure S1: Association between the recurrence rate and the follow-up duration, with the associated Pearson correlation coefficient R and p-value; Figure S2: Funnel plot showing the distribution of studies comparing the recurrence rate among low vs. high WHO grade spinal meningiomas; Figure S3: Funnel plot showing the distribution of studies comparing the recurrence rate among ventral vs. non-ventral spinal meningiomas; Figure S4: Funnel plot showing the distribution of studies comparing the recurrence rate spinal meningiomas operated with Simpson grade 1 vs. grade 2 resection; Figure S5: Funnel plot showing the distribution of studies comparing the recurrence rate spinal meningiomas operated with Simpson grade 1 and 2 vs. grade 3, 4, and 5 resections; Table S1: Baseline characteristics and study inclusion under each of the sections of this review; Table S2: Risk of bias assessment; Table S3: Epidemiology; Table S4: Histopathology; Table S5: Genetics and immunohistochemistry; Table S6: Tumor location; Table S7: Presenting symptoms; Table S8: Non-surgical treatment options; Table S9: Surgical treatment of spinal meningniomas; Table S10: Intraoperative neuromonitoring; Table S11: Perioperative complications; Table S12: Neurological outcomes; Table S13: Markers of neurologic outcomes as described by the included studies; Table S14: Recurrence rate and markers of recurrence.
