# Supplementary material for: Current Knowledge on Spinal Meningiomas—Surgical Treatment, Complications, and Outcomes: A Systematic Review and Meta-Analysis (Part 2)
Source: Cancers (Basel). 2022 Dec 16;14(24):6221. doi: 10.3390/cancers14246221 (PMC9777510; doi:10.3390/cancers14246221)
Supplement: Supplementary file 1 [file cancers-14-06221-s001.zip › Supplementary file S2, Search strategy.pdf]

Database	Queries
PubMed	((("spinal meningioma*"[Title] NOT "case report"[All Fields]) AND 2000/01/01:3000/12/31[Date - Publication] AND "english"[Language] AND ("loattrfull text"[Filter] AND "humans"[MeSH Terms] AND "english"[Language])) OR ((("spin*"[All Fields] AND ("meningioma"[MeSH Terms] OR "meningioma"[All Fields] OR "meningiomas"[All Fields])) NOT ("case reports"[Publication Type] OR "case report"[All Fields])) AND 2000/01/01:3000/12/31[Date - Publication] AND ("loattrfull text"[Filter] AND "humans"[MeSH Terms] AND "english"[Language]))) AND ((fft[Filter]) AND (humans[Filter]) AND (english[Filter]))
Web of Science	((ALL=("spinal meningioma") AND PY=(2000-2500) AND LA=(English) NOT TS=("CASE REPORT")) NOT (TASCA==("VETERINARY SCIENCES") OR DT==("EDITORIAL MATERIAL" OR "LETTER" OR "MEETING ABSTRACT"))) OR ((TI=(spinal AND meningioma*) AND PY=(2000-2500) AND LA=(English) NOT TI=(case report)) NOT (TASCA==("VETERINARY SCIENCES") OR DT==("EDITORIAL MATERIAL" OR "LETTER" OR "MEETING ABSTRACT")))
Embase	spinal meningioma*':ab,ti NOT 'case report' AND [2000-2021]/py NOT ('spinal meningioma*':ab,ti NOT 'case report' AND [2000-2021]/py) AND ('animal experiment'/de OR 'animal model'/de OR 'animal tissue'/de OR 'nonhuman'/de)
